# Visual search habits and the spatial structure of scenes

**DOI:** 10.3758/s13414-022-02506-2

**Published:** 2022-07-11

**Authors:** Alasdair D. F. Clarke, Anna Nowakowska, Amelia R. Hunt

**Affiliations:** 1 Department of Psychology, University of Essex, Colchester, UK; 2 Department of Psychology, University of Aberdeen, Aberdeen, UK

**Keywords:** Visual search, Optimal behaviour, Eye movements

## Abstract

Some spatial layouts may suit our visual search habits better than others. We compared eye movements during search across three spatial configurations. Participants searched for a line segment oriented 45^∘^ to the right. Variation in the orientation of distractor line segments determines the extent to which this target would be visible in peripheral vision: a target among homogeneous distractors is highly visible, while a target among heterogeneous distractors requires central vision. When the search array is split into homogeneous and heterogeneous left and right halves, a large proportion of fixations are “wasted” on the homogeneous half, leading to slower search times. We compared this pattern to two new configurations. In the first, the array was split into upper and lower halves. During a passive viewing baseline condition, we observed biases to look both at the top half and also at the hetergeneous region first. Both of these biases were weaker during active search, despite the fact that the heterogeneous bias would have led to improvements in efficiency if it had been retained. In the second experiment, patches of more or less heterogeneous line segments were scattered across the search space. This configuration allows for more natural, spatially distributed scanpaths. Participants were more efficient and less variable relative to the left/right configuration. The results are consistent with the idea that visual search is associated with a distributed sequence of fixations, guided only loosely by the potential visibility of the target in different regions of the scene.

## Introduction

There is a well known joke in which a a policeman stops to help a drunk man search for his keys under a street-light. After searching for a few minutes the policeman asks the drunk if he is sure he lost them here. The drunk replies, no, and that he lost them in the park. When the policeman asks why he is searching under the street-light, the drunk replies, “this is where the light is”. This behaviour has been refereed to as the *street-light effect* or *drunkard’s search* and has been used as metaphor in social and behavioural sciences since at least the 1960s (Kaplan, 1964).

**Fig. 1:**
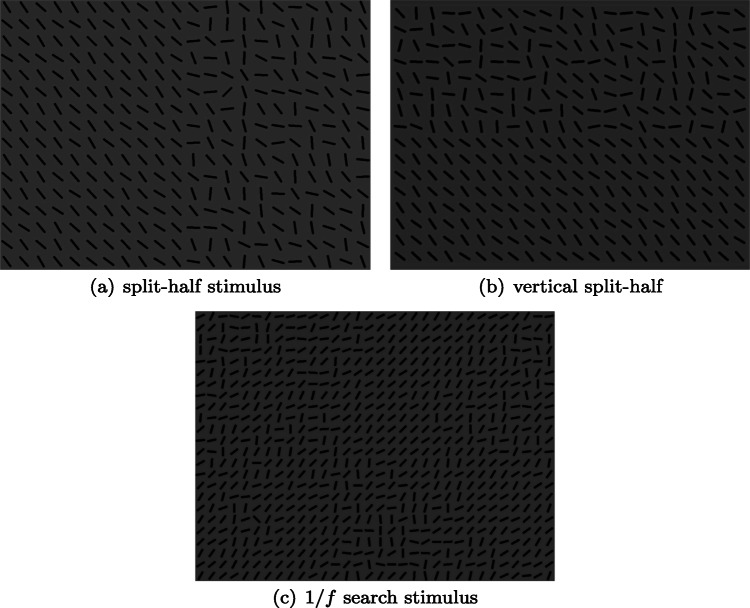
(a) Example stimulus from Nowakowska et al., (2017), reused in Experiment 2 of the present study. (b) The rotated, vertical split-half stimulus used in Experiment 1. (c) Experiment 2 contrasted search strategies in split-half stimuli with these new 1/*f* stimuli. Please note that in this experiment we used a denser array of line segments for all conditions. Both classes of stimuli consist of a mix of hetero- and homogeneous line segments

We have observed that this is is also a good description of how some observers approach a complex visual search task (Nowakowska, Clarke, & Hunt, 2017). In this task, observers were faced with an array of oriented line segments (Fig. 1(a)) and had to report if a unique target was present or not. When faced with such a search task the optimal strategy is to direct your attention to the half of the display containing the heterogeneous line segments: if the target was present on the homogeneous side, then it would be easy to see with peripheral vision and no further searching would be required. This experiment was originally carried out to investigate whether human observers follow this optimal strategy (Najemnik & Geisler, 2008) or adopt stochastic search behaviour (Clarke, Green, Chantler, & Hunt, 2016). While we found that the group average behaviour was in line with predictions of the stochastic search model, closer inspection revealed a large range of individual differences, with some observers near-optimal, and others following the same strategy as the drunkard in the above joke.

A similar range of individual differences in visual search have been reported by Irons and Leber (2016) using their *attentional control* paradigm, in which there are two targets of different colours, and participants choose which target to search for. There are varying numbers of distracters with colours matching one or other of the two targets, with the optimal strategy being to search for the target that matches the fewest number of distracters. This paradigm has been shown to have good test-retest reliability (*r* ≈ 0.8) (Irons & Leber, 2018). Clarke, Irons, James, Leber and Hunt (2022b) tested participants in both the split-half and attentional control paradigms and found no correlations between the two tasks, either in terms of strategy or mean reaction time. In addition, participants also completed a mouse-tracking version of the conjunction foraging task developed by Kristjánsson, Jóhannesson and Thornton (2014), which also showed no correlation with the other two paradigms. However, the original results of all three paradigms replicated, and the split-half visual search task was shown to have similar test-retest reliability (*r* ≈ 0.75) to that of the attentional control paradigm. Clearly, the individual differences found with these different paradigms must be driven by different aspects of the specific task and stimulus.

One salient difference between the stimuli across these paradigms is the spatial distribution of distracters. The spatial layout of scenes has become increasingly well-established as an important factor in determining the sequential selection of regions for closer inspection, both in terms of deciding where to fixate (e.g. Henderson, Weeks, & Hollingworth, 1999) and also in the speed of detecting targets (e.g. Castelhano and Heaven (2011). Familiar, repeated layouts have long been known to implicitly guide attention (Chun & Jiang, 1998), leading to faster target detection relatively to unfamiliar search contexts. These various findings all converge on the idea that scene layout is a “guiding feature”. That is, many aspects of spatial layout can be processed in the absence of attention and can therefore guide the sequential selection of items or locations for focused attention and/or fixation (Wolfe, 2021).

In the present study, we investigate the role of spatial layout in determining the range of individual differences observed in the split-half visual search task. It is clear that human observers make use of a number of heuristics, biases and preferences that are independent of the visual scene currently under inspection. A specific spatial layout may be more or less compatible with spatial biases in visual exploration. One particularly strong example is the central bias (Tatler & Vincent, 2009) which is when observers preferentially fixate the centre of an image. Other biases that have been documented in the literature include coarse-to-fine strategies (Over, Hooge, Vlaskamp, & Erkelens, 2007) and a bias to make saccades to the left of the display (Nuthmann & Matthias, 2014). These effects are robust and have been replicated in many different studies, especially the central bias (Clarke & Tatler, 2014) which offers a better prediction of fixation distribution than many traditional salience models (Kümmerer, Wallis, & Bethge, 2018).

A related concept is that of cognitive strategies, such as left-to-right and top-to-bottom scan patterns (Gilchrist & Harvey, 2006). Rather than employ a guided search (looking for items that share features with the target) or optimal search (moving the eyes to the location that will maximise the chance of then finding the target), such strategies use a pre-set “plan” that allows the observer to work their way methodically through the search display with little cognitive overhead. A similar idea is the saccadic flow model by Clarke, Stainer, Tatler and Hunt (2017) in which an observer makes saccades at random until the target is found. Such stochastic strategies have been shown to offer human-like performance in some conditions (Clarke et al., 2016). However, when we consider the split-half search stimuli, it is clear that such strategies will fail to provide good performance and lead to equal numbers of fixations on either side of the display. Dividing the search array vertically into easy and hard halves is not a “natural” division; the artificial nature of the search scene may be misaligned with typical search behaviour.

To explore this hypothesis we present a variation of the original split-half visual search task. In the first experiment, we simply split the stimuli horizontally (as opposed to vertically), to create an upper and a lower search region. This was motivated by the observation that in our everyday environment, the upper visual field is usually uncluttered, i.e., the sky or ceiling, compared to the ground-plane. By rotating the boundary between the two halves of our stimuli, we make them more similar to the natural world, and this potentially increases the chance that our participants can use a familiar heuristic that approximates the optimal search strategy, by letting the scene implicitly guide them to the locations that are more informative. If so, we also would expect improved performance when the heterogeneous half of the display is in the lower region than when it is in the upper region. We also included a passive viewing phase to this experiment, to measure the extent to which people distribute their fixations over the stimulus differently when they are actively searching for a specified target compared to when they are asked to simply look at the same stimulus.

In the second experiment, we smoothly vary the heterogeneity of the distracters across the search space to create a random distribution of heterogeneous and homogeneous regions. This would allow participants to efficiently scan the search array using a more natural and familiar distribution of saccadic amplitudes and directions. Across both experiments, the key measure of search strategy is the proportion of fixations landing on the heterogeneous parts of the array. We exclude the first fixation because it is always at screen center. We include only up to fixation 6 on each trial because, while the number of fixations made on each trial varies a great deal, almost all trials have at least six fixations. We use the data only from target absent trials in calculating this proportion to ensure all fixations are associated with searching for the target (rather than finding or identifying it). We have shown previously that the proportion of heterogeneous-side fixations during the early part of target absent trials is correlated strongly with how quickly targets are found when they are present (Nowakowska et al., 2017; Nowakowska, Clarke, von Seth, & Hunt, 2021).

## Experiment 1: Horizontal split array

In everyday life we are accustomed to more cluttered (difficult) search areas occurring in the lower visual field, and the objects we tend to search for on a daily basis (keys, pens, bank cards) tend to be in the lower visual field. Here we divide the search array into upper and lower fields (as opposed to the left/right division used in our previous experiments such as Nowakowska et al. (2017, 2021). If the spatial structure of the visual scene facilitates efficient search, we should see better performance, particularly when the hard (heterogeneous) search is in lower visual field.

The methods and planned analysis for this study were registered on the Open Science Framework^1^ before data collection started.

### Methods

#### Participants

16 participants (11 females) took part in the Experiment 1 (median age= 25, age range = 22 − 49). The participants were recruited from the student community at the University of Aberdeen. All participants had normal or corrected to normal vision and provided informed consent. The experiment was approved by the University of Aberdeen Psychology Ethics Committee. The sample size is similar to previous research using this paradigm (Nowakowska et al., 2017), where *n* = 14 was sufficient to show a wide range of differences between participants.

#### Apparatus

Experimental scripts were created and run using MatLab with psychophysics (Brainard, 1997) with the Eyelink (Cornelissen, Peters, & Palmer, 2002) toolboxes. The experiment was displayed on a 17-inch CRT monitor with a resolution of 1024 × 768. Participants placed their heads in a chin rest for the duration of the experiment and responses were recorded using a standard keyboard. Eye movement were tracked monocularly tracked using an EyeLink 1000 eye tracker in the desktop configuration (SR Research, Canada).

#### Stimuli

Stimuli consisted of an array, 22 columns and 16 rows, of black line segments displayed on a uniform grey background. The target was a line segment oriented 45^∘^ to the right. The non-target (distractor) line segments had a random orientation, with a mean angle perpendicular to the target. An example is shown in Fig. 1(b). On one half (upper or lower) of the array, the variance of the distractor orientation was narrow (*π*/6), which is referred to below as the “easy” or “homogeneous” half. On the other half of the array, the variance of the distractor orientation was wider (3*π*/3). We refer to this as the “hard” or “heterogeneous” half. In pilot experiments (see Nowakowska et al., 2017) in which the display duration was too short to permit any eye movements, detection performance was at ceiling on the easy background, and at chance on the hard background. This supports our assumption that participants should direct their eye movements to the hard half of the array when they are split into hard and easy halves.

#### Procedure

Participants were seated in a dimly lit room using a chin rest set 50cm in front of the monitor. A nine-point calibration was completed prior to beginning each block. The researcher was present in the room for each calibration. The experiment began with ten passive viewing trials, in each of which the search array was presented for 5 s. Participants were only told “We will show you a series of images. We would like you to view these images.” These trials were included to assess where in the search array participants were drawn to look when no search target had been specified. Following this, the search target was identified and the response keys explained, and 5 practice trials were completed. The participant was then left alone in the room to complete each of four blocks of experimental trials, with the experimenter re-entering to calibrate the participant between blocks. So as not to distract the participant, the experimenter was monitoring the participant through a window in the door. Participants were told to respond as quickly and accurately as possible. Before each trial participants were required to fixate the center of a fixation cross then press any key to begin. Trials would not begin unless participants were fixating the cross. Participants reported if the target was present (TP) or absent (TA) using the up (present) and down (absent) arrow keys. The target was present on half the trials. Each array was presented until either the participant made a response or timed out after 60s. Visual feedback was provided for an incorrect response in the form of a red screen. Each participant completed 40 trials in which the target was present on the easy half of the display, and 40 in which it was present on the hard half of the display. In 80 trials the target was absent. These were randomly intermixed. Stimulus configuration (whether the hard side was presented on the top or the bottom half of the display) varied randomly from trial to trial.

#### Preprocessing

Fixations landing outside of the search area were coded as NaN and not included in analysis. Fixations were classed as falling on the homogeneous or heterogeneous half of the display, with those landing on the central 30-pixel horizontal strip being left unclassified. The vast majority of the first fixations are, by this definition, unclassified because the trial is only initiated when the participant fixates the center. We therefore only analyse fixations 2–6. 89*%* of second fixations are classed as either homogeneous or heterogeneous, and this rises to 95*%* for later saccades. The measure of search strategy (referred to as efficiency) is the proportion of fixations on the heterogeneous side out of the the total fixations classed as either heterogeneous or homogeneous, with 1 being perfect efficiency. We only use target absent trials when we calculate search strategy, to avoid including fixations that are directed towards the target itself.

#### Analysis

The eye-tracking data was processed with the default SR Research parser to give sequences of fixations and saccades. All other data analysis was done in Rv3.4.0 (R. Core Team, 2017) with the tidyverse collection of packages. Bayesian generalised multi-level models were fitted using brms (Bürkner, 2017) with four chains of 4000 iterations each. A maximal random effects structure was used, with weakly informative priors. All data and analysis scripts are available here.^2^

### Results

#### Accuracy and reaction time

**Fig. 2:**
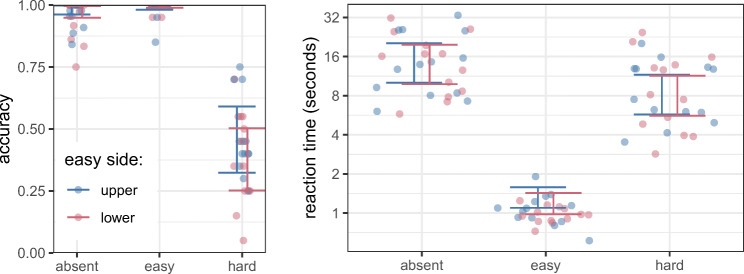
The points show each individual participant’s (left) accuracy and (right) median reaction time. The error bars indicate the 95*%* HPDI interval for the average participant. Please note that the *y*-axis on the right hand graph is on a log scale. Reassuringly, there is no evidence of a spatial compatibility effect arising from the use of the up and down keys for participants’ responses

Three participants were removed due to low (< 75%) accuracy in the *target absent* condition. The accuracy of the remaining 13 participants is shown in Fig. 2 (left) along with the HPDI of a Bayesian generalized mixed effect model.^3^ In general, accuracy in the target absent was near 100*%*, as was accuracy when the target was present on the easy half of the display. In contrast, a little under half of the targets on the hard side of the display were found. The spatial configuration, whether the easy side was at the top of bottom of the stimulus, had negligible effect on accuracy. In the remaining analysis for this experiment, only trials with correct responses are considered. Figure 2(right) shows the reaction time data. We can see that *easy* targets are found quickly, typically in around 1 s, while *hard* targets and TA trials take much longer (around 8 and 15 s respectively).

#### Search strategy

**Fig. 3:**
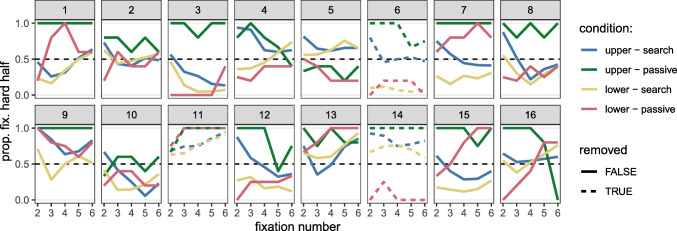
Saccadic data visualised for the visual search and passive viewing conditions. Each subplot corresponds to an individual participant. The lines show the proportion of trials in which each fixation was on the heterogeneous (hard) half of the display, for fixations 2–6 on target absent trials only with optimal being a proportion of 1. The results replicate the wide range of individual differences seen in Nowakowska et al., (2017) and Clarke et al., (2022b). Particpants 6, 11 and 14 were removed from all analyses for failing to meet pre-specified criteria, but are shown here for full reporting of all data collected

Figure 3 shows the saccadic search strategies for our observers, expressed as the proportion of fixations 2–6 that were directed to the heterogeneous side (on target absent trials only). We can see that as expected, there are large individual differences in the efficiency score during visual search, with some approaching an optimal strategy (e.g., participant 11); some fixating each half of the display evenly (participants 16 & 2); and others following a counter-optimal strategy (participant 3). There are also large differences in viewing behaviour in the passive viewing condition, although intriguingly, there is no clear link between a participant’s behaviour in the two tasks.

**Fig. 4:**
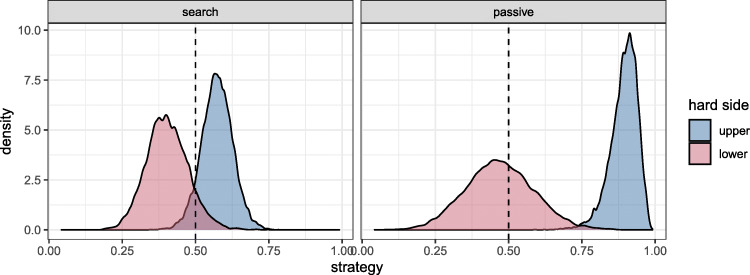
Posterior distribution for the proportion of fixations to the hetero- (hard) and homogeneous (easy) halves of the display (plotting as “strategy” on the *x*-axis). The plots show the effect of spatial configuration (whether the hard side is placed in the upper or lower half of the display) for the two tasks (search on the left and passive on the right)

To investigate this in more detail, we fitted a multi-level generalized linear model to the fixation data to explore how the probability of fixating the heterogeneous side of the display varied with spatial configuration and task. We can see from Fig. 4 that both factors appear to have an effect. In the search condition, on average, participants show at best a small preference to fixate the heterogeneous side of the display when it is presented above the homogeneous side (95*%* HDI = [0.416,0.631]), and the opposite behaviour when the spatial configuration is reversed ([0.259,0.519]). (The HDI for the difference between distributions is [0.053,0.229].) This essentially shows that observers have a slight preference to fixate the upper half of the display. The pattern is quite different in the passive viewing condition: When the heterogeneous side of the display is in the lower configuration, participants fixate both sides evenly (HDI [0.247,0.701]). However, when the heterogeneous side is presented above the homogeneous side, participants show a strong preference to look at it for all of the first fixations ([0.762,0.973]). This pattern is consistent with a preference to fixate the heterogeneous side during passive viewing, in addition to a preference to fixate the upper half.

### Discussion

The results from this study replicate the large individual differences in search strategy demonstrated by Nowakowska et al., (2017). Our measure of search strategy (focusing on the fixations 2–6 on target absent trials) revealed an overall slight tendency towards the efficient strategy (i.e. to fixate the heterogeneous side), but there were also many inefficient fixations made on the homogeneous side of the search array, and a wide range of difference in the extent to which individual participants did this. We did not see the hypothesised increase in search efficiency when the more heterogeneous part of the scene was in the lower visual field. What emerged instead was a small preference to search the upper side of the search display initially, which translated to higher search efficiency when the heterogeneous side was in the upper visual field. Surprisingly, we see a much larger effect of spatial configuration in the passive viewing condition, in which the majority of our observers (at least 7 out of 13) fixated the upper region of the display almost exclusively for the fixations we analysed (2–6), as long as this region was heterogeneous. When the bottom was heterogeneous, participants were roughly equally likely to fixate either half. It is important to note that there are only ten trials of passive viewing and they came at the beginning of the experiment, before the participants were informed of the task, the target, and the response associated with the search task. We cannot rule out the possibility that it is exposure to the stimulus, rather than search per se, that is responsible for the changes in participants’ viewing behaviour when shifting from the passive to the search condition. Nonetheless, one possible explanation for poor search behaviour that is made less plausible by the passive viewing results is that participants simply prefer the homogeneous side as more pleasing or restful to view, considering that their preference for the homogeneous side is even lower when they are not searching.

We can conclude there is a bias to fixate the upper field first. This bias is strengthened when the upper field is heterogeneous, but this is not specific to search; indeed, active searching for a specified target reduces, rather than increases, the tendency to fixate the heterogeneous regions when they appear in the upper half of the search array.

## Experiment 2: Jumbled search arrays

In our second experiment, we investigate how search in a jumbled mix of hetero- and homogeneous regions differs from a split-half array. At first glance, it seems like it will be harder to execute an efficient sequence of saccades through these stimuli. There is no longer a clear texture boundary between the easy and hard halves of the display, and staying fixated on those regions where central vision is needed requires a far more complex sequence of saccades than when the region comprises one large contiguous block. On the other hand, the optimal search strategy for the split-half stimuli is trivially easy to implement, at least, at the level at which we analyse the scan paths. Nonetheless, most of our participants fail to implement it. The more complex nature of the jumbled search array may in fact facilitate more optimal search strategies, as the heterogeneous patches are now spread out throughout the scene, allowing an observer to target them while also making use of a more natural and familiar search heuristic.

### Methods

#### Participants

34 participants^4^ took part in the experiment.^5^ Eight participants were removed from the analysis due to low accuracy on either the target absent trials (< 75%, indicating that they did not understand the task or were confused about the target) or hard trials (< 25% indicating that they had adopted a strategy of not even trying to find the less salient targets). All participants had normal or corrected-to-normal vision, provided informed consent, and were remunerated *£*7.50 for their time.

#### Apparatus

Experimental scripts were created and run using MatLab with psychophysics (Brainard, 1997) with the Eyelink (Cornelissen et al., 2002) toolboxes. The experiment was displayed on a monitor with a resolution of 1280 × 1024. Participants heads were placed in a chin rest for the duration of the experiment and responses were recorded using a keyboard. Eye movements were tracked monocularly with a desktop EyeLink 1000.

#### Stimuli

The variance of the distracters varied across four conditions: *easy*, *hard*, *split-half* and *jumbled*. (Note: we used the original left-right configuration for the split-half stimuli, not the up-down.) If the target was present, then its location was placed at random in the array, with the constraint that it could not appear along the edge of the stimulus, or be one of the four central line segments. In this study we used a denser array of line segments than Nowakowska et al., (2017) (32 × 24 compared to 22 × 16) in order to allow for more complexity and variation in the *jumbled* stimuli. For *easy* trials, the distracters all had a similar orientation, allowing for the target (if present) to be identifiable with peripheral vision. In the *hard* condition the line segments were much more heterogeneous, making the target hard to find. *Split-half* trials consisted of homogeneous line segments one side of the search array, and heterogeneous on the other.

**Fig. 5:**
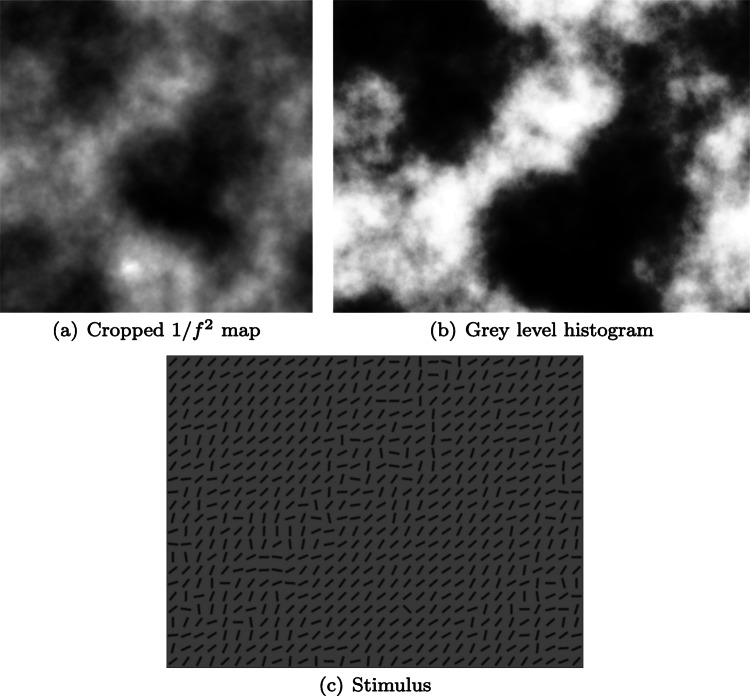
Illustration of the stages involved in creating the stimuli

The *jumbled* stimuli were created in two stages. First of all a 1024 × 1024 pixel array of 1/*f*^2^-noise (with random phase) was generated. (These dimensions were chosen as powers of two work well with Fast Fourier Transforms.) This array was then truncated to match the dimensions of the desired search array (1024 × 768), before the grey levels were histogram equalised to a reference distribution. This was done to ensure that each stimulus had the same distribution of difficulties. We used a parabola as the reference distribution in order to set the majority of pixels to low or high regions and create distinctive homo- and heterogeneous regions in the search array (Fig. 5).^6^

The search array is created by tiling 32 × 32 pixel squares, each containing a line segment. The line segment orientations are randomly sampled from a uniform distribution centred on *𝜃* + *π*/2 (where *𝜃* is the orientation of the target in the current block) and range determined by the 1/*f*^2^-map described above. A value of 0 in this difficulty map corresponds to a small range of *π*/6 while a value of 1 gives a range of 2*π*/3.

#### Procedure:

Before the experiment each participant was given a nine-point calibration sequence and practice trials. Participants were instructed they should identify if the target is present or absent as quickly and accurately as possible. Each trial began with a central fixation point on a blank grey screen. To start each trial participants were required to fixate on the centre point and press space-bar. Participants had to press either the F (absent) or the J (present) key. Feedback was given in the form of the screen flashing red and a beep if the given response was incorrect.

There were 100 trials in total broken down into four blocks. Conditions were mixed and randomised within a block. Participants were given the opportunity to rest and the eye tracker was re-calibrated in between each block.

### Results

#### Accuracy

**Fig. 6:**
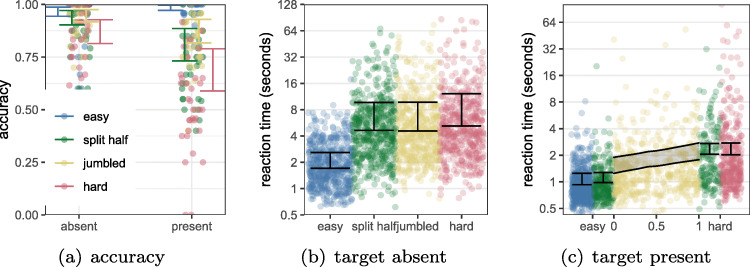
(a) Mean accuracy for each condition over participants. (b, c) Reaction times for each of the four stimulus conditions. Each point represents a trial, and the error bars and shaded region indicate the 95*%* HPDI of the effects estimated from a Bayesian generalized mixed effects model. Reaction times for TP *split-half* and *jumbled* trials have been broken down by the distractor difficulty at the location of the target, which can range from 0 (easy) to 1 (hard)

Mean accuracy is shown in Fig. 6(a).^7^ As expected, accuracy for the target absent trials was high for all four conditions (details). Mean accuracy for target present trials was 98*%* for *easy* trials, compared to 68*%* for *hard* targets. Unsurprisingly, accuracy for the *split-half* and *jumbled* trials depended on the target’s location. For the *split-half* stimuli, mean accuracy for the easy and hard half of the display was 98*%* and 61*%*, i.e., very similar to trials with a uniformly easy or hard search array. For the *jumbled* trials, mean accuracy ranged from 91*%* for targets located in a region with distractor difficulty less than 0.1, dropping to 63*%* for regions with a difficulty above 0.9. Incorrect trials were not included in the analysis below.

#### Reaction times

Figure 6(b,c) shows that the reaction times follow the expected pattern: when the target is located in the easy half of the *split-half* array, this is as difficult to find as a target in an *easy* trial, and similar for the hard side. We can see that observers give up searching for a target after approximately 6 s in the *hard*, *split-half* and *jumbled* conditions, compared to the 2 s required to search the *easy* stimuli. However, there is a high degree of variability in each condition. In the *jumbled* condition, reaction times increase with distractor difficulty, although we do see a slight range compression: a distractor difficulty of 0 is slightly harder than the *easy* trials, while a difficulty of 1 is easier than the *hard* trials. This is likely due to the easier and harder regions being relatively small compared to the *easy*, *hard* and *split-half* stimuli. Full details of the Bayesian model used to summarise the data can be found in the Supplementary Materials.

#### Search strategy

**Fig. 7:**
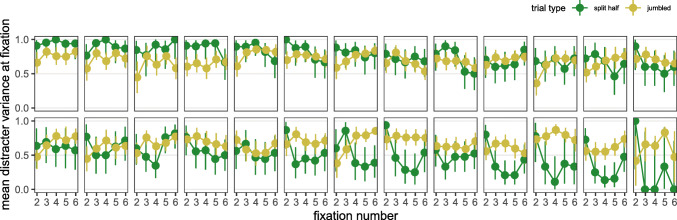
Saccadic search strategies for each participant. Values closer to 1 indicate that participants were directing their saccades to heterogeneous regions of the search array, and hence searching more optimally. Participants have been ordered by how optimal their strategy was

Following Nowakowska et al., (2017), and the same as in Experiment 1, search strategies for the *split half* stimuli were defined as the proportion of fixations (*n* = 2,…6) directed to the heterogeneous half of the search display during target absent trials. As can be seen in Fig. 7, we again replicate the findings of Nowakowska et al., (2017), with large individual differences for the *split half* stimuli: During the initial part of a search trial, some participants are close to optimal, some search both sides equally, while others show a counter-optimal strategy. This is in contrast to the *jumbled* condition where we can see that our participants all show a preference for fixating the heterogeneous regions of the display.

#### Correlations between conditions

**Fig. 8:**
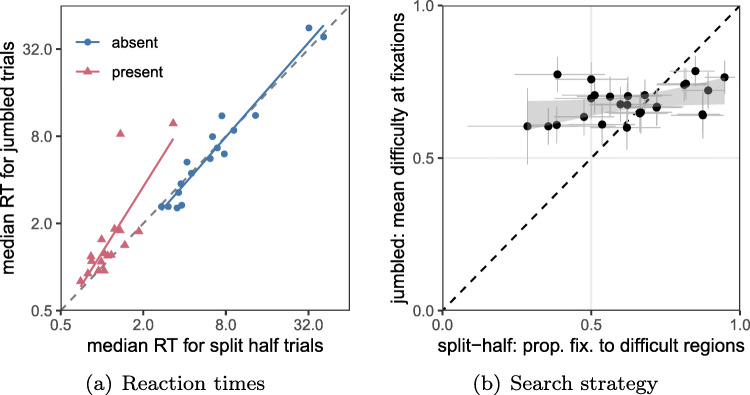
(a) Correlation between reaction times in the *split-half* and *jumbled*. There is very little difference in median RT between the two conditions. This is especially true for target absent trials. (b) Individual differences in eye movement strategies. While there are large differences from one individual to the next in the *split half* condition, there is much less variation for *jumbled* stimuli

As can be seen in Fig. 8(a), there is a strong correlation between ($\log _{2}$) median reaction times in the *split half* and *jumbled* conditions (*R*^2^ = 0.92 and 0.50 for TA and TP respectively. The full correlation matrix is given in Table 1). Individuals are remarkably consistent in terms of RT between the *split-half* and *jumbled* stimuli. However from Fig. 8(b), it is clear that the variability in terms of their eye-movement data is restricted, resulting in a low correlation (*r* = 0.39, 95*%CI* = [0.005,0.676]) between the *split half* and *jumbled* conditions in terms of the proportion of saccades to heterogeneous regions.

**Table 1:** Correlation matrix for $\log _{2}$ reaction times in Experiment 2. Values above 0.9 have been marked in bold

	Target absent				Target present			
	Easy	Hard	Split half	Jumbled	Easy	Hard	Split half	Jumbled
Easy	1	0.32	0.48	0.33	1	0.19	0.42	0.21
Hard	0.32	1	**0.94**	**0.97**	0.19	1	0.63	**0.91**
Split half	0.48	**0.94**	1	**0.94**	0.42	0.63	1	0.53
Jumbled	0.33	**0.97**	**0.94**	1	0.21	**0.91**	0.53	1

### Discussion

Overall, we successfully replicated the wide range of saccadic search strategies seen in Nowakowska et al., (2017) and Clarke et al., (2022b). For the *jumbled* condition, we found far less variation: Despite the more complex segregation into homo- and hetero-geneous regions when compared with the *split half* stimuli, all of our participants consistently directed their saccades towards the more heterogeneous regions of the display.

## Discussion

We replicated the large individual differences seen in how human observers search *split half* stimuli, and extended this to a condition where the screen was split into upper and lower halves, as well for a split between left and right halves. However, for the *jumbled* stimuli, in which the heterogeneous regions were distributed across the search area, scanning behaviour is much more consistent with observers showing a preference to direct saccades to the more heterogeneous regions of the search array. Why are observers able to use distractor heterogeneity to guide their search for the *jumbled*, but not for *split half* arrays, when it would be more beneficial? One explanation is that in order to efficiently search the *split half* arrays, an observer has to override their default search strategy (Clarke and Tatler, 2014; Clarke et al., 2016, 2017; Gilchrist & Harvey, 2006; Amor, Luković, Herrmann, & Andrade, 2017) to scan a subregion of the array. This is not the case for the *jumbled* stimuli: observers can target the heterogeneous regions while still following their usual search dynamics.

Previous experiments showing a wide range of mostly poor search strategies (Nowakowska et al. 2017, 2021) used a display in which the screen was split vertically into left and right halves. This configuration bears little resemblance to how information is distributed in most natural scenes. Given the importance of scene layout in guiding search (e.g. Wolfe, 2021; Zinchenko, Conci, Töllner, Müller, & Geyer, 2020), in the current experiments we asked whether more familiar or natural layouts might improve search strategies. The horizontal-split configuration used in Experiment 1 more closely matches many familiar scenes, particularly when the array is high-variance on the bottom half and low-variance on the top (Torralba & Oliva, 2003). In Experiment 2, the jumbled array of high-variance and low-variance patches allows the participant to fixate the information-rich regions that were scattered around the whole scene, rather than restrict themselves to a particular side. We hypothesised that these more natural, familiar configurations might encourage more uniformly optimal search among participants, because their habits and biases are no longer at odds with the spatial configuration of the display. The results across the two experiments were mixed, with the vertical split condition closely replicating the poor strategies seen with the left/right split. The patchy array, however, did encourage more uniformly optimal search, despite that fact that implementing an optimal strategy is arguably more difficult in this condition.

Participants tend to fixate the top part of the display early in each trial. This bias was overall stronger than the preference for the easy or hard side, at least for fixations in aggregate (some individual participants had a stronger bias towards the hard or easy side). The tendency to explore the upper part first during passive viewing could be related to physiological structure of oculomotor muscles, or to the way we tend to scan text during reading. This natural tendency to scan from top to bottom seems to be weakened during active search.

Similarly, but more surprisingly, the passive viewing condition also revealed a clear preference to fixate the heterogeneous half early in the trial. This was seen in the strong preference to fixate the upper half when it was heterogeneous, and a weaker preference for the upper half when it was homogeneous. Thus, the passive view condition shows a systematic pattern of favouring both the hard side and the upper field. This demonstrates that participants are sensitive to the differences between the halves and prefer to fixate the more cluttered, information-rich part of the scene, a bias which should have served participants well during active search, by directing fixations to the regions where central vision is needed. This makes it particularly surprising that introducing a search task and defining the target made this tendency to fixate the heterogeneous side weaker instead of stronger.

These results suggest that different mechanisms govern the allocation of fixations, depending on task. Without a specific directive to search (and also early in the experiment, given that passive viewing trials were always shown first), the upper half and the heterogeneous half attracted more fixations. Defining a search target and giving participants a task weakened both the tendency to fixate the upper half, as well as the tendency to fixate the heterogeneous side of the search array.

In the introduction we speculated that restricting the information in the search array to one contiguous half of the array could, on the one hand, make it easier to identify and focus on this half and ignore the other. On the other hand, this could be an unnatural and unfamiliar division that might work against the fixation strategies we use during active search that are adapted to the statistics of our typical visual environments. The results from the jumbled search array support this latter idea by showing that distributing the heterogeneous regions around the display made participants more, rather than less, efficient. The range of individual differences was also much narrower with the jumbled than the split-half stimuli, suggesting a more consistent bias towards the heterogeneous parts of the array. This improvement stands in stark contrast to other manipulations to this task that have not led to improved efficiency. Specifically, Nowakowska et al., (2021) varied both the time constraints (untimed versus a 2 s deadline), and reward (a financial incentive to improve reaction time in a second block of trials) and found that neither manipulation improved efficiency. A key difference between that study and this one is that here we have increased efficiency by manipulating the context to better suit the natural search behaviour of the participants. Changing the search behaviour of the participants to suit the context appears to be a more challenging undertaking.

The results of these experiments also reinforce our previous argument that group-level effects and individual differences must be complementary pieces of a larger understanding of visual search (Clarke, Nowakowska, & Hunt, 2019). The average behaviour of participants searching for the target when the screen is divided in half (whether horizontally or vertically) would suggest no clear preference for the heterogeneous or homogeneous side, but these averages hide clear preferences for one or the other among some individual participants. We have shown previously that these differences are reliable over time (Nowakowska, Clarke, Sahraie, & Hunt, 2019; Nowakowska et al., 2021), making them a useful starting point for investigation using a correlational approach. On the other hand, the behaviour of participants in the jumbled task is more convergent, making the average efficiency score for this specific condition a reasonable representation of the group’s behaviour. This same similarity across individuals, however, is what limits the conclusions that can be drawn from correlational analyses (or more specifically, from a lack of such correlations). This distinction between reliable effects of a manipulation on a group and reliable individual differences is summarized nicely by Hedge, Powell and Sumner (2018). The role of spatial layout in driving the individual differences observed in foraging (Kristjánsson et al., 2014) noted in the introduction is an interesting question for future research to explore. Recent efforts to measure both spatial and target selection biases in foraging (Clarke, Hunt, & Hughes, 2022a) offers the potential for new insights into how and when observers use spatial layout to guide sequential target selections.

While the majority of observers are clearly not actively implementing an optimal search strategy in the *split half* condition, the results from the *jumbled* condition suggest that observers can search through arrays of oriented line segments efficiently under some conditions. This is consistent with previous work showing that that human behaviour during search is comparable to both the ideal observer (Najemnik and Geisler, 2008) and a random walk (Clarke et al., 2016). Perhaps the stochastic search strategy is honed by evolution and/or experience to facilitate near-optimal behaviour when allowed for by the structure of the environment. While some form of active control is required to account for the results of Experiment 2, this could be explained by a simple saliency effect in which observers follow a stochastic search strategy while favouring the more heterogeneous regions. The absence of an overall optimal strategy in the split-half versions of the search array in both Experiment 1 and in Experiment 2 argue against a prediction of information gain being the primary mechanism of fixation selection during search, as suggested by the ideal search model. If it were, the split-half condition should have only made it easier to predict which regions would yield the most new information.

In conclusion, the results demonstrate that eye movements during search follow distributed patterns, and are not easily guided by information to remain confined to particular regions of the search area. This tendency to broadly distribute fixations seems to be particularly prevalent during search, relative to passive viewing.

## Footnotes

1 https://osf.io/8qgju/

2 https://osf.io/8qgju/

3 Full details given in supplementary materials

4 The sample size is larger than for Experiment 1 because this experiment was designed in part to measure the correlation in search efficiency across the two spatial configurations.

5 Sadly, demographic information such as age range and gender split have been lost, but are likely to be similar to Experiment 1. These data are irrelevant for our analysis plan

6 MatLab code available from OSF

7 Accuracy for each person is shown in Figure 2.1 of the *supplementary materials*
